# Archaeal community diversity and abundance changes along a natural salinity gradient in estuarine sediments

**DOI:** 10.1093/femsec/fiu025

**Published:** 2014-12-15

**Authors:** Gordon Webster, Louise A. O'Sullivan, Yiyu Meng, Angharad S. Williams, Andrea M. Sass, Andrew J. Watkins, R. John Parkes, Andrew J. Weightman

**Affiliations:** 1 Cardiff School of Biosciences, Cardiff University, Main Building, Park Place, Cardiff, Wales, CF10 3AT, UK; 2 School of Earth and Ocean Sciences, Cardiff University, Main Building, Park Place, Cardiff, Wales, CF10 3AT, UK

**Keywords:** *Archaea*, methanogens, estuarine sediment, salinity gradient, pyrosequencing, qPCR

## Abstract

*Archaea* are widespread in marine sediments, but their occurrence and relationship with natural salinity gradients in estuarine sediments is not well understood. This study investigated the abundance and diversity of *Archaea* in sediments at three sites [Brightlingsea (BR), Alresford (AR) and Hythe (HY)] along the Colne Estuary, using quantitative real-time PCR (qPCR) of 16S rRNA genes, DNA hybridization, *Archaea* 16S rRNA and *mcr*A gene phylogenetic analyses. Total archaeal 16S rRNA abundance in sediments were higher in the low-salinity brackish sediments from HY (2–8 × 10^7^ 16S rRNA gene copies cm^−3^) than the high-salinity marine sites from BR and AR (2 × 10^4^–2 × 10^7^ and 4 × 10^6^–2 × 10^7^ 16S rRNA gene copies cm^−3^, respectively), although as a proportion of the total prokaryotes *Archaea* were higher at BR than at AR or HY. Phylogenetic analysis showed that members of the ‘Bathyarchaeota*’* (MCG), *Thaumarchaeota* and methanogenic *Euryarchaeota* were the dominant groups of *Archaea*. The composition of *Thaumarchaeota* varied with salinity, as only ‘marine’ group I.1a was present in marine sediments (BR). Methanogen 16S rRNA genes from low-salinity sediments at HY were dominated by acetotrophic *Methanosaeta* and putatively hydrogentrophic *Methanomicrobiales*, whereas the marine site (BR) was dominated by *mcr*A genes belonging to methylotrophic *Methanococcoides*, versatile *Methanosarcina* and methanotrophic ANME-2a. Overall, the results indicate that salinity and associated factors play a role in controlling diversity and distribution of *Archaea* in estuarine sediments.

## Abstract (precis)

*Archaea* populations in the River Colne Estuary, UK sediments change with increasing salinity gradient from populations dominated by methanogenic *Euryarchaeota* and ‘*Bathyarchaeota*’ (MCG) in brackish sediments to putatively ammonia-oxidising *Thaumarchaeota* and MCG in marine sediments.

## Graphical abstract

**Figure:**
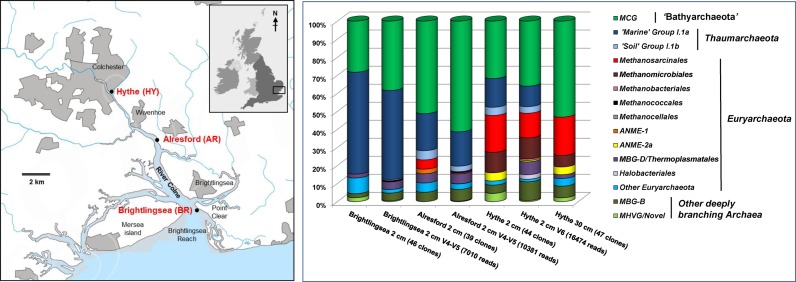
*Archaea* populations in the River Colne Estuary, UK, sediments change with increasing salinity gradient from populations dominated by methanogenic *Euryarchaeota* and ‘Bathyarchaeota’ (MCG) in brackish sediments to putatively ammonia-oxidizing *Thaumarchaeota* and MCG in marine sediments.

## INTRODUCTION

Estuaries are semi-enclosed coastal bodies of water where rivers meet the sea, and because estuaries are interfaces between riverine and marine habitats, they are extremely dynamic, with steep physico-chemical gradients due to variability of freshwater input, geomorphology and tidal heights (Meire *et al.*, 2005; Bernhard and Bollmann 2010). Characteristically, estuaries exhibit strong gradients along their course with organic matter and nitrogen concentrations normally decreasing away from the estuary head, and chloride and sulphate increasing towards the estuary mouth. The resulting gradients in salinity, turbidity, nutrients and organic matter influence the composition of the estuarine prokaryotic community (Crump *et al.*, 2004; Freitag, Chang and Prosser 2006; Bernhard and Bollmann 2010), and this community, in turn, is critical in controlling the function and structure of estuarine ecosystems (Day *et al.*, 1989). Since estuaries tend to have high concentrations of nutrients, they exhibit elevated primary production and heterotrophic activity that result in high levels of microbial activity in the upper sediment layers, which subsequently generate steep biogeochemical gradients with depth (Canfield and Thamdrup 2009). Therefore, sedimentary *Bacteria* and *Archaea* play an important role in the dynamics of estuarine environments, particularly in biogeochemical cycles and food webs. However, relatively little is known about the diversity of the estuarine sediment prokaryotic community, and in particular the *Archaea*.

The application of molecular techniques to study microbial ecology over the last two decades has completely changed our perception of the diversity, distribution and function of *Archaea* in natural marine ecosystems. Analysis of 16S rRNA gene sequences from many environmental samples has revealed that *Archaea* are ubiquitous (e.g. DeLong 1992; Stein and Simon 1996; Schleper, Jurgens and Jonuscheit 2005; Wuchter *et al.*, 2006; Kubo *et al.*, 2012; Vila-Costa *et al.*, 2013) and far more abundant than previously assumed (Karner, DeLong and Karl 2001; Cavicchioli 2011). Molecular phylogenetic approaches have revealed the existence of novel *Archaea* lineages within the open-ocean, subsurface and coastal marine sediments, soils and freshwater lakes (DeLong 1992; Jurgens *et al.*, 2000; Ochsenreiter *et al.*, 2003; Webster *et al.*, 2006, 2010; Kubo *et al.*, 2012; Lloyd *et al.*, 2013b). These mesophilic *Archaea*, belonging to the *Euryarchaeota*, *Thaumarchaeota* (Brochier-Armanet *et al.*, 2008) and the recently proposed ‘Bathyarchaeota’ formerly known as Miscellaneous Crenarchaeotal Group (MCG) (Meng *et al.*, 2014), are now recognized to be widespread in marine sediments and reported to contribute significantly to carbon and nitrogen cycling within these environments (Francis *et al.*, 2005; Ingalls *et al.*, 2006; Parkes *et al.*, 2007; Knittel and Boetius 2009; Lloyd *et al.*, 2013b; Meng *et al.*, 2014). For example, pure culture representatives and laboratory enrichments include species that are able to carry out methanogenesis, anaerobic methane oxidation and ammonia oxidation (Könneke *et al.*, 2005; Liu and Whitman 2008; Knittel and Boetius 2009; Watkins *et al.*, 2012).

The River Colne Estuary on the east coast of the UK is a macrotidal, hypernutrified, muddy estuary with strong gradients of dissolved organic carbon (DOC), nitrate and ammonium decreasing from the estuary head to the estuary mouth (Dong *et al.*, 2000; Thornton *et al.*, 2002). To date, studies on prokaryotic diversity in this estuary have mainly focused on *Bacteria* involved in nitrification, nitrate reduction, denitrification and sulphate reduction (e.g. Dong *et al.*, 2000; Nogales *et al.*, 2002; Purdy *et al.*, 2003; Nedwell, Embley and Purdy 2004; Smith *et al.*, 2007; Li *et al.*, 2014), with some studies focused on methanogenic *Archaea* (Munson, Nedwell and Embley 1997; Purdy *et al.*, 2002; Oakley *et al.*, 2012; O'Sullivan *et al.*, 2013). However, little is known about the overall archaeal community at this site with respect to an estuarine salinity gradient and related conditions.

Molecular analyses of *Archaea* from temperate and tropical (Abreu *et al.*, 2001; Vieira *et al.*, 2007; Zeng, Li and Jiao 2007 Webster *et al.*, 2010; Kubo *et al.*, 2012; Lazar *et al.*, 2014) estuaries indicate that estuarine sediments contain a diverse population of novel *Archaea*, possibly as a consequence of the presence of both freshwater and coastal ocean populations (Singh *et al.*, 2010; Xie *et al.*, 2014). In view of the limited information on the effect of estuarine salinity gradients on *Archaea*, we have examined archaeal abundance and diversity, and compared phylogenetic relationships at different sediment sites from the same estuary using culture-independent 16S rRNA and *mcr*A gene analyses. This study expands previous investigations into the prokaryotic diversity of the Colne Estuary, Essex, UK, and specifically investigated *Archaea* and methanogen diversity in contrasting sediments along a salinity gradient. In addition, it complements the study by O'Sullivan *et al.* (2013) on the application of 16S rRNA gene PCR-DGGE to investigate the bacterial and archaeal community structure along the Colne Estuary.

## METHODS

### Site description, sediment sampling, cell counts and chemical analysis

**Figure 1.**
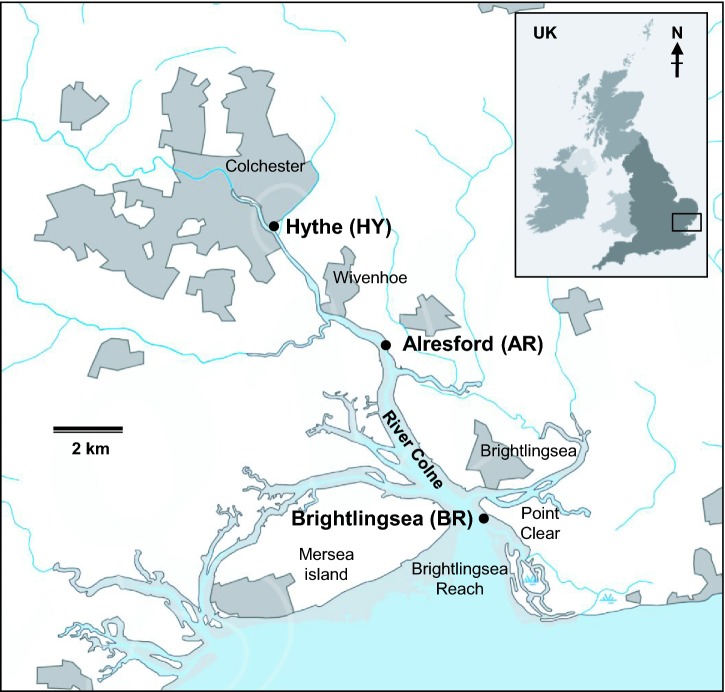
Location of the River Colne Estuary, Essex, UK, sediment sampling sites, BR, AR and HY. The Colne Estuary, Scale 1:200 000 [PDF map], OS Strategi, Ordnance Survey UK, updated January 2011. Map created September 2011 using EDINA Digimap Ordnance Survey Service, http://edina.ac.uk/digimap.

Triplicate sediment cores (10 cm diameter, up to 60 cm depth) were collected from three sites along the River Colne Estuary, Essex, UK, in October 2005 (O'Sullivan *et al.*, 2013). Sample sites (Fig. 1) were Brightlingsea (BR), an open mud creek at the estuary mouth (51°47.920^′^N, 01°01.075^′^E); Alresford (AR), a mid-estuary creek (51°50.716^′^N, 00°58.912^′^E); Hythe (HY), a salt marsh at the estuary head (51°52.687^′^N, 00°56.011^′^E). Sediment cores were sealed with rubber stoppers, transported to the laboratory under cooled conditions on ice and cores for molecular analysis were sub-sampled within 4 h of collection. One core per site was sub-sampled aseptically into 2 cm-depth sections and the middle of each section transferred to a sterile 50 ml volume plastic tube and stored at −80°C until required for DNA extraction. Sediment samples were also preserved in serum vials containing filter-sterilized (0.2 μm) 4% (v/v) formaldehyde in artificial seawater and prokaryotic cells were enumerated by acridine orange direct count (AODC) method (O'Sullivan *et al.*, 2013).

The remaining sediment cores for chemical analysis were stored for up to 7 days at 4°C, prior to sub-sampling. Cores were aseptically sectioned into 2 cm-depths and sub-sampled with sterile 5 ml syringes (luer end removed). Pore waters were obtained from sediments by centrifugal extraction (Webster *et al.*, 2010) and sulphate and chloride concentrations determined using an ICS-2000 Ion Chromatography System with two Ionpac AS15 columns in series and an Anion Self-Regenerating Suppressor (ASRS-ULTRA II 4 mm) in combination with a DS6 heated conductivity cell (Dionex UK Ltd., Camberley, UK) as described (Webster *et al.*, 2009). Salinity was calculated from chloride values using the formula: S‰ = 0.03 + 1.805 × Cl‰ (Carritt 1963). Sediment for methane gas analysis was transferred into 20 ml volume serum vials with 10 ml of 10% (w/v) KCl, sealed and stored at 20°C overnight for equilibration. Headspace gas was analyzed using a Perkin Elmer/Arnel Clarus 500 natural gas analyser with a flame ionization detector and a thermal conductivity detector.

### DNA extraction

Genomic DNA was extracted from sediment samples using the FastDNA Spin Kit for Soil (MP Biomedicals, Solon, OH, USA) as described (Webster *et al.*, 2003; O'Sullivan *et al.*, 2013). Duplicate DNA extractions were performed on sediment samples down to 30 cm depth (0–2, 4–6, 8–10, 12–14, 16–18, 20–22, 24–26 and 28–30 cm) for each site, pooled and purified using Microcon centrifugal filters (Merck Millipore Ltd., Cork, Ireland) and eluted in 100 μl sterile molecular grade water (Severn Biotech Ltd., Kidderminster, UK).

### Quantitative real-time PCR

Quantitative real-time PCR (qPCR) was used to quantify 16S rRNA gene copy numbers of *Bacteria*, *Archaea* and *Methanococcoides* species in sediment samples with depth. SYBR Green chemistry was used for all protocols. All qPCR reactions for standards, no template controls and sediment samples were undertaken in triplicate and run on an Agilent Mx3000P QPCR System (Agilent Technologies UK Ltd., Stockport, UK). For standard curves and calibration, serial dilutions of full length 16S rRNA gene PCR products (Table 1) from *Anaerolinea thermophila* DSM 14523 and *Methanococcoides methylutens* DSM 2657 were used as standards for *Bacteria* and *Archaea*, and *Methanococcoides* species. To ensure good quantification data, qPCR results were rejected if the R^2^ value of the standard curve was below 0.95 or the efficiency of the reaction was not between 90 and 110%. The qPCR mixtures for all reactions (standards, controls and samples) were contained in a total volume of 20 μl with 400 nM of each primer (Eurofins MWG Operon, Ebersberg, Germany), 2 μg bovine serum albumin (BSA; Promega, Southampton, UK) and 1 μl of DNA in 1x qPCRBIO SyGreen Lo-ROX Mix (PCR Biosystems Ltd., London, UK) made up with molecular grade water (Severn Biotech Ltd.). 16S rRNA gene primers 534F/907R (Muyzer, De Waal and Uitterlinden 1993; Muyzer *et al.*^1998^) and S-D-Arch-0025-a-S-17F/S-D-Arch-0344-a-S-20R (Vetriani *et al.*, 1999) were used to target the *Bacteria* and *Archaea*, respectively (Table 1). The protocol was 95°C for 7 min, 40 cycles of 95°C for 30 s, 52°C for 30 s, 72°C for 60 s, followed by a melting curve from 55 to 95°C. Each cycle was followed by data acquisition at the elongation step. *Methanococcoides*-specific qPCR was carried out with primers designed using Primer3Plus (Untergasser *et al.*, 2007) that amplify a 147 bp product; primers were designated Mc416F and Mc524R (Table 1). The *Methanococcoides*-specific qPCR protocol was essentially as above, but with an annealing temperature of 60°C.

**Table 1.** Oligonucleotide primers and probes used in this study.

Primer/probe	Target gene	Sequence (5^′^-3^′^)	Reference	Approach
109F	*Archaea* 16S rRNA	ACK GCT CAG TAA CAC GT	Grosskopf, Janssen and Liesack (1998)	PCR
958R	*Archaea* 16S rRNA	YCC GGC GTT GAM TCC AAT T	DeLong (1992)	PCR
ME1f	*mcr*A	GCM ATG CAR ATH GGW ATG TC	Hales et al. (1996)	PCR
ME2r	*mcr*A	TCA TKG CRT AGT TDG GRT AGT	Hales et al. (1996)	PCR
MLf	*mcr*A	GGT GGT GTM GGA TTC ACA CAR TAY GCW ACA GC	Luton et al. (2002)	PCR
MLr	*mcr*A	TTC ATT GCR TAG TTW GGR TAG TT	Luton et al. (2002)	PCR
27F	*Bacteria* 16S rRNA	AGA GTT TGA TCM TGG CTC AG	Lane (1991)	qPCR standard
1492R	*Bacteria* 16S rRNA	GGT TAC CTT GTT ACG ACT T	Lane (1991)	qPCR standard
534F	*Bacteria* 16S rRNA	GCC AGC AGC CGC GGT AAT	Muyzer, De Waal and Uitterlinden (1993)	qPCR
907R	*Bacteria* 16S rRNA	CCG TCA ATT CCT TTG AGT TT	Muyzer et al. (1998)	qPCR
A8f	*Archaea* 16S rRNA	CGG TTG ATC CTG CCG GA	Lepage et al. (2004)	qPCR standard
A1492r	*Archaea* 16S rRNA	GGC TAC CTT GTT ACG ACT T	Teske et al. (2002)	qPCR standard
S-D-Arch-0025-a-S-17F	*Archaea* 16S rRNA	CTG GTT GAT CCT GCC AG	Vetriani et al. (1999)	qPCR
S-D-Arch-0344-a-S-20R	*Archaea* 16S rRNA	TC GCG CCT GCT GCG CCC CGT	Vetriani et al. (1999)	qPCR
Mc416F^a^	*Methanococcoides* 16S rRNA	ATG TTG GCT GTC CAC ATG TG	This study	qPCR
Mc524R^a^	*Methanococcoides* 16S rRNA	CCG AAG AAC TGA TCA AAC CG	This study	qPCR
P958-DIG^b^	*Archaea* 16S rRNA	DIG-YCC GGC GTT GAM TCC AAT T-DIG	DeLong (1992)	DNA hybridization
P915-DIG^b^	*Archaea* 16S rRNA	DIG-GTG CTC CCC CGC CAA TTC CT-DIG	DeLong (1992)	DNA hybridization
P355-DIG^b^	*Methanosarcinales/Methanomicrobiales* 16S rRNA	DIG-CAG GCG CGA AAA CTT TAC-DIG	Banning et al. (2005)	DNA hybridization
PmcrA-DIG^b,c^	*mcr*A	DIG-GGT GGT GTM GGA TTC ACA CAR TAT GCA ACA GC-DIG	This study	DNA hybridization

^a^Based on *M. burtonii* 16S rRNA gene numbering.

^b^DIG, oligonucleotide probe labeled at both 5^′^ and 3^′^ end with digoxigenin. Note specific formamide (%; hybridization) and NaCl (M; washing) concentrations used for each probe were: 20% and 0.19 M for P958, 30% and 0.074 M for P915, 50% and 0.019 M for P355, 30% and 0.074 M for PmcrA.

^c^Modified from Luton et al. (2002).

### *Archaea* 16S rRNA and *mcr*A gene PCR conditions

*Archaea* 16S rRNA genes (covering variable regions V2–V5) were amplified from selected sediment DNA extracts [BR 0–2 cm depth (BR2), AR 0–2 cm depth (AR2), HY 0–2 cm depth (HY2), HY 28–30 cm depth (HY30)] using primers 109F/958R as described (O'Sullivan *et al.*, 2013; Table 1). Methanogen-specific methyl-coenzyme M reductase (*mcr*A) genes were amplified by nested PCR using primers ME1f/ME2r (Hales *et al.*, 1996) and MLf/MLr (Luton *et al.*, 2002) as described (O'Sullivan *et al.*, 2013; Table 1). *Archaea* PCR mixtures were contained in a total volume of 50 μl with 200 nM each primer (Eurofins MWG Operon), 2 U *Taq* DNA polymerase (Promega), 1.5 mM MgCl_2_, 0.2 mM each dNTP (Promega), 10 μg BSA (Promega) and 1 μl DNA template in 1x PCR buffer (Promega) made up with molecular grade water (Severn Biotech Ltd.). Reaction mixtures for *mcr*A were as above, except 3 mM MgCl_2_ was added, and all second round nested PCRs were performed without BSA. PCR set up was carried out under aseptic conditions with autoclaved and/or UV-treated plasticware and pipettes. Amplifications were with a Dyad DNA Engine thermal cycling machine (MJ Research, Waltham, MA, USA). All sets of PCRs included appropriate positive (*Methanoplanus petrolearius* DSM 11571) and negative (molecular grade water) controls.

### 16S rRNA and *mcr*A gene library construction and DNA hybridization

Five replicate PCR products for each sample were cleaned, pooled and cloned in pGEM-T Easy vector and transformed into *Escherichia coli* JM109 competent cells (Promega) according to manufacturer's protocol. Recombinant colonies (384 colonies for 16S rRNA and 192 colonies for *mcr*A gene libraries) were picked for each sample and grown overnight at 37°C in 96-well plates containing LB liquid medium with 7.5% (v/v) glycerol and 100 μg ml^−1^ ampicillin, and the libraries stored at −80°C. Clones (5 μl) were spotted onto positively charged nylon membranes (Roche Diagnostics Ltd., Burgess Hill, UK) and allowed to air-dry before treatment with denaturing solution [0.5 M NaOH, 1.5 M NaCl, 0.1% (w/v) SDS]. Membranes were then neutralized (1 M Tris-HCl, 1.5 M NaCl, pH 7.5), washed with 2x SSC solution (0.3 M NaCl, 0.03 M sodium citrate, pH 7) and the DNA bound to the membrane by UV crosslinking. Finally, membranes were air-dried and stored at room temperature until required for hybridization.

All archaeal 16S rRNA and *mcr*A gene library membranes were screened with 5^′^ and 3^′^ end-labeled digoxigenin (DIG) oligonucleotide probes (Eurofins MWG Operon) targeting *Archaea* and methanogen-specific 16S rRNA genes and *mcr*A genes (Table 1), respectively, under optimized conditions. Membranes were treated with pre-hybridization solution [5x SSC, 2% (w/v) blocking reagent (Roche Diagnostics Ltd.), 0.1% (w/v) N-lauroylsarcosine and 0.02% (w/v) SDS] for 1 h at 46°C in a hybridization oven (Stuart Scientific, Chelmsford, UK). Hybridization was then carried out by treating each membrane with hybridization solution [5x SSC, 4% (w/v) blocking reagent, 0.1% (w/v) N-lauroylsarcosine, 0.01% (w/v) SDS, 20 ng ml^−1^ DIG-labeled probe, 20–50% (v/v) deionized formamide (concentration depending on probe; Table 1)] at 46°C for 16–18 h. Membranes were then washed twice with stringency wash solution [0.01% (w/v) SDS, 0.02 M Tris-HCl, 0.019–0.19 M NaCl (concentration depending on probe; Table 1), pH 7.4] for 15 min each at 46°C. Chemiluminescent detection of the hybridized probe was carried out by first equilibrating the membrane for 1 min in maleic acid buffer [0.1 M maleic acid, 0.3% (v/v) Tween 20, 0.15 M NaCl, pH 7.5] before blocking for 30–60 min in 1% (w/v) blocking reagent in maleic acid buffer. After blocking, the membrane was incubated in a 1:10 000 dilution of anti-digoxigenin antibody conjugated to alkaline phosphatase (Roche Diagnostics Ltd.) for 30 min. Membranes were washed free of unbound antibody by washing the membrane twice, 15 min per wash, in maleic acid buffer before incubation with a 1:100 dilution of CSPD chemiluminescent substrate diluted in detection buffer (0.1 M NaCl, 0.1 M Tris-HCl, pH 9.5) and incubated for 15 min at 37°C. Detection of the chemiluminescent signal was undertaken by exposure of the membrane to Kodak BioMax XAR film for 1–16 h at room temperature, and films were developed using Kodak GBX developer/replenisher.

### Phylogenetic analysis

Approximately 30 to 50 recombinant clones from each *Archaea* 16S rRNA or *mcr*A gene library, identified by DNA hybridization, were amplified by PCR with M13 primers and sequenced using an ABI 3130xl Genetic Analyzer (Applied Biosystems, Foster City, CA, USA). Sequence chromatographs were analyzed using the Chromas Lite software package version 2.01 (http://www.technelysium.com.au/). Sequences were checked for chimeras with Bellerophon software (Huber, Faulkner and Hugenholtz 2004) and searched for sequence similarities in databases using nucleotide BLAST analysis (Altschul *et al.*, 1990). Sequences were assigned to various operational taxonomic units (OTUs) or phylotypes by using BLASTClust (http://www.ncbi.nlm.nih.gov/) at 95 and 97% similarity for 16S rRNA gene sequences and 89% for *mcr*A gene sequences, representing suggested genus and species level groupings (Schloss and Handelsman 2004; Steinberg and Regan 2008). Statistical parameters including rarefaction curves, library coverage (Good's coverage), Shannon's and Simpson's indices of diversity, species richness (*S*_Chao1_) and abundance-based coverage estimator (*S*_ACE_) values were calculated using the Past software package version 2.08b (Hammer, Harper and Ryan 2001) and the web interface of Kemp and Aller (2004).

All 16S RNA gene sequences were aligned using ClustalX (Thompson *et al.*, 1997) with sequences retrieved from the database. Alignments were edited manually using BioEdit Sequence Alignment Editor version 7.1.3 (Hall 1999) and regions of ambiguous alignment were removed. The phylogenetic relationships between pairs of 16S rRNA gene sequences were determined using distance and implemented in MEGA4 (Tamura *et al.*, 2007). The LogDet distance analysis (Lockhart *et al.*, 1994) constructed using minimum evolution was used as the primary tool for estimating phylogenetic relationships, but other methods including *p*-distance and Jukes–Cantor with minimum evolution and neighbor joining were also carried out, which yielded similar tree topologies. All distance trees were bootstrapped 1000 times to assess support for nodes.

New sequences reported here have been submitted to the EMBL database under accession numbers HG001325-HG001413 for 16S rRNA gene sequences and HG001414-HG001452 for *mcr*A gene sequences.

### 16S rRNA gene tag sequencing

Variable regions 4 and 5 (V4–V5) of the 16S rRNA gene from *Archaea* were amplified from DNA from BR (0–2 cm depth, BR2) and AR (0–2 cm depth, AR2) using barcoded fusion primers A519F/A958R, and 454 pyrosequencing was performed on a Roche 454 GS FLX+ at ChunLab, Inc., Seoul, Korea. All PCR methods, primers and analysis tools are detailed on the ChunLab website (http://www.chunlab.com). Further analysis of sequencing data was performed in QIIME version 1.7.0 (Caporaso *et al.*, 2010) using a pipeline developed ‘in house’ at Cardiff University (D.A. Pass *et al.* unpublished data). Essentially, all sequence files were checked using Acacia software release 1.53 (Bragg *et al.*, 2012) for quality, sequence errors and to reduce noise. Representative OTUs were picked with UCLUST (Edgar 2010) at 97 and 94% similarity and taxonomy assigned using BLAST (Altschul *et al.*, 1990) with the Greengenes database (DeSantis *et al.*, 2006). Singletons and non-specific sequences (e.g. *Bacteria* sequences) were then removed and diversity estimates were calculated in QIIME at 97 and 94% similarity.

DNA extracted from HY (0–2 cm depth, HY2) sediment was also amplified using methods of the International Census of Marine Microbes (ICoMM). The V6 region of the 16S rRNA gene from *Archaea* was also amplified and subjected to 454 pyrosequencing on a GS20. All PCR methods, primers and analysis tools are detailed on the ICoMM website (http://vamps.mbl.edu/; Sogin *et al.*, 2006). The clusters were generated using the single-linkage pre-clustering algorithm to smooth sequencing errors and reduce noise, followed by primary pairwise, average linkage clustering. OTUs were created using clustering thresholds of 3 and 6%, corresponding to 97 and 94% similarity, respectively (Huse *et al.*, 2010). Further analysis of the dataset was carried out using QIIME version 1.2.1 (Caporaso *et al.*, 2010). Tag sequences are publicly available from ICoMM (http://vamps.mbl.edu/) as the dataset CFU_0012_2006_10_25.

## RESULTS AND DISCUSSION

### Sediment pore water sulphate, methane and salinity

There were clear differences in sediment pore water chemistry between the three sediment sampling sites, which corresponded to their location within the River Colne Estuary (Figs 1 and 2). For example, at 2 cm sediment depth, sulphate and chloride concentrations were at their highest (∼28 and 530 mM, respectively) at the estuary mouth (BR, marine sediment; Fig. 2a) and at their lowest (∼7 and 130 mM, respectively) at the estuary head (HY, brackish sediment; Fig. 2c) corresponding to a salinity range of 34.5 to 8.4‰. While at the mid-sampling point (AR), sulphate, chloride and salinity values (∼25, 460 mM and 29.9‰, respectively) were slightly lower than at BR. These values are consistent with previous data at or near to the sample locations used (Dong *et al.*, 2000; Thornton *et al.*, 2002), and demonstrate a clear salinity gradient along Colne Estuary sediments. However, salinity values at HY can vary temporally due to the influence of high tides (Thornton *et al.*, 2002), although pore water salinities are always within the range for brackish sediments. Similarly, near surface sulphate concentrations also show variation due to tidal and seasonal influences (Purdy *et al.*, 2003; Nedwell, Embley and Purdy 2004).

**Figure 2.**
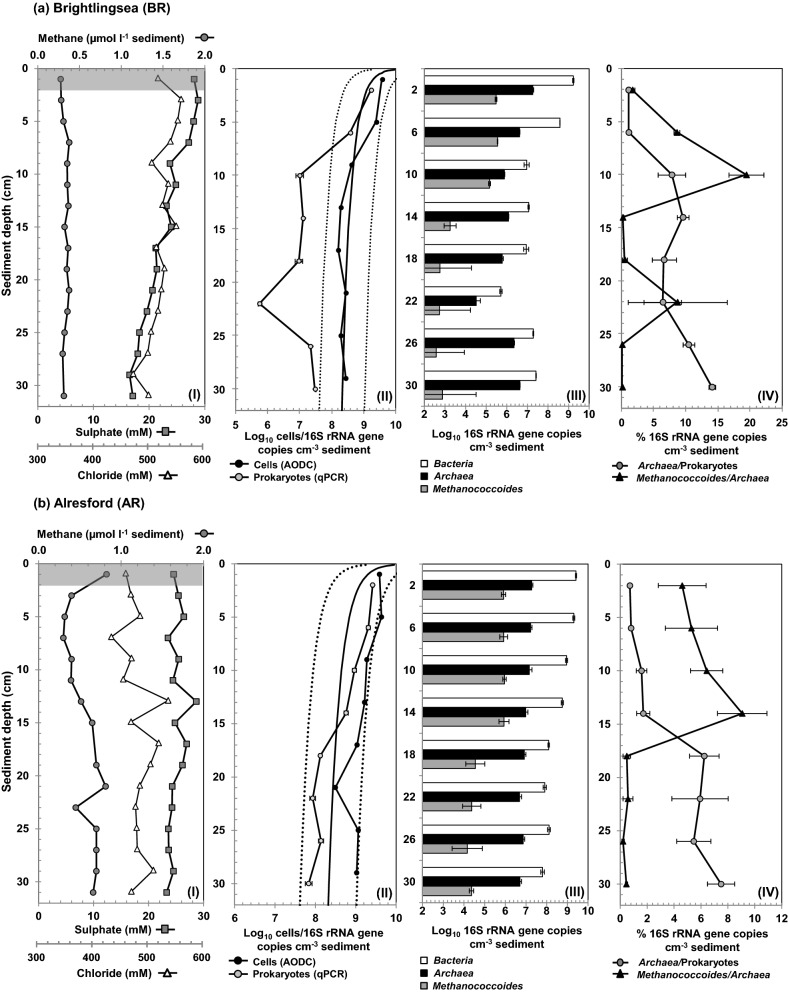
Depth profiles of geochemical data, total cell numbers and *Archaea* 16S rRNA gene copies for Colne Estuary sediment cores, (a) BR, (b) AR and (c) HY. Graph panels show data for (I) pore water chloride, sulphate and methane; shaded region denotes depths of samples used for archaeal 16S rRNA and *mcr*A gene libraries. (II) Log10 total cell numbers determined by AODC and prokaryotic 16S rRNA gene copy numbers determined by qPCR. The solid line shows Parkes, Cragg and Wellsbury (2000) general model for prokaryotic cell distributions in marine sediments, and dotted lines represent 95% prediction limits. (III) Log10 16S rRNA gene copy numbers for *Bacteria*, *Archaea* and *Methanococcoides* species. (IV) Percentage of *Archaea* and *Methanococcoides* species of the total prokaryotic and *Archaea* populations, respectively. All qPCR data points are means of three replicates.

**Figure 2.**
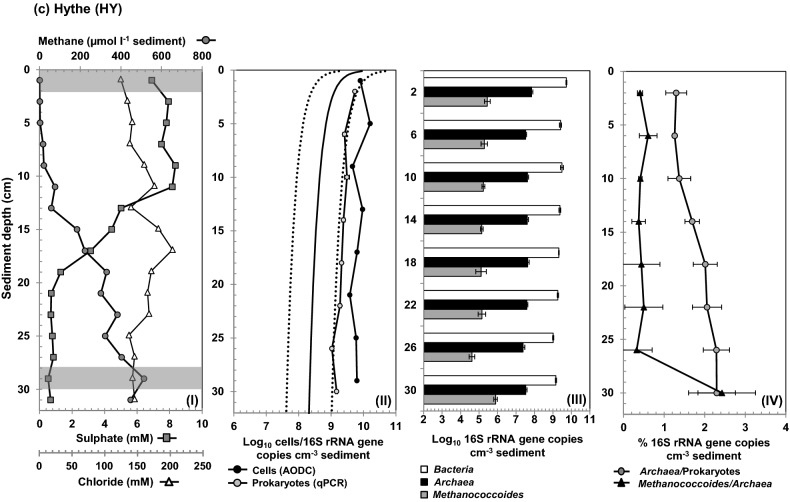
*Continued*.

At all three sampling sites, concentrations of sulphate decreased with sediment depth; at HY, it decreased steeply down to 0.7 mM at ∼20 cm (Fig. 2c), whereas the marine sites (BR and AR) had lower rates of sulphate removal, reaching ∼17–22 mM at 30 cm (Fig. 2a and b). Concentrations of methane were also low at BR and AR (<1 μmol l^−1^ sediment) and remained as such throughout the depth analyzed, while concentrations of methane at HY increased rapidly from ∼1 μmol l^−1^ sediment at the surface to >500 μmol l^−1^ sediment at 30 cm, with a broad sulphate–methane transition zone between 10 and 20 cm where sulphate and methane profiles intersected (Fig. 2c). Previously, Nedwell, Embley and Purdy (2004) also reported low rates of methane formation in surface (top 20 cm) sediments of the Colne Estuary, with no discernible trend in relation to site location; however, samples for that study were from a limited range of depths and it was predicted that methane formation would be elevated in low sulphate deeper sediments (Nedwell, Embley and Purdy 2004), as at HY in this study (Fig. 2c).

Additional geochemical data [temperature, volatile fatty acids (VFAs)] and methanogenic activity for these sites were previously reported (O'Sullivan *et al.*, 2013; Fig. S1, Supporting Information). In summary, sediment temperatures in October 2005 were slightly higher at HY (15°C) than at AR or BR (∼14°C), and VFAs (acetate, lactate and formate) concentrations were consistently low (<43 μM) at all sediment depths, with the highest concentrations of VFAs being measured at AR (Fig. S1, Supporting Information). Rates of methanogenesis (O'Sullivan *et al.*, 2013) were generally low at BR and AR at all depths analyzed (e.g. 20.8 and 2.3 pmol cm^−3^ d^−1^, respectively at 2 cm), whereas at HY rates were low at the surface 2 cm (2.9 pmol cm^−3^ d^−1^) and then increased with depth (e.g. 104 pmol cm^−3^ d^−1^ at 30 cm depth), consistent with the high methane concentrations at HY (Fig. 2c). However, the rates in the study by O'Sullivan *et al.* (2013) were 100 to 1000-fold lower than those previously estimated for the Colne Estuary using sediment methane production (Purdy *et al.*, 2002; Nedwell, Embley and Purdy 2004), but are comparable with earlier ^14^C-tracer experiments at Colne Point salt marsh (Senior *et al.*, 1982). Previous studies also show that Colne Estuary sediments decrease in concentration of dissolved organic nitrogen, ammonium and organic carbon as salinity increases towards the estuary mouth (Dong *et al.*, 2000), ranging from ∼0.3 to >0.1 mM, ∼1 to 0.05 mM and ∼4 to 1%, respectively (Thornton *et al.*, 2002; Agedah *et al.*, 2009).

### Total prokaryotic cell counts, *Bacteria* and *Archaea* 16S rRNA gene copy numbers

Cell counts (AODC) at all sites decreased with depth and followed the global trend (Parkes, Cragg and Wellsbury 2000) for marine sediments; cell counts at HY (brackish sediment) were substantially higher (Fig. 2) than BR and AR, possibly due to high nutrient input at the estuary head (Dong *et al.*, 2000). qPCR of DNA copy numbers of total prokaryotic 16S rRNA genes (sum of bacterial and archaeal 16S rRNA gene qPCR counts) were generally lower (∼5–10 fold) than the AODC (Fig. 2), with the exception of all surface sediments, which only differed slightly (∼2 fold). However, despite this, at all three sites AODC and prokaryotic 16S rRNA gene copy number were in good agreement, with an overall decrease in cell/copy numbers with depth, as well as higher numbers of prokaryotic 16S rRNA genes being detected at HY than at BR or AR. Such discrepancies in cell numbers between qPCR and AODC data have been reported previously and using a meta-analysis of several data sets Lloyd *et al.* (2013a) demonstrated that in sediments qPCR measurements are poorly predicted by total cell counts, even after accounting for variations in 16S rRNA gene copy number per genome. However, qPCR measurements were relative to other qPCR data from the same samples and it was concluded that qPCR was a reliable relative quantification method (Lloyd *et al.*, 2013a). Similarly, in our study both archaeal and bacterial 16S rRNA gene copy numbers generally decreased with depth, and *Bacteria* were the dominant prokaryotic group at all sites and depths (86–99% of total prokaryotes; Fig. 2).

Despite the apparent bacterial dominance, *Archaea* constituted a substantial part of the Colne Estuary sediment community. Total archaeal 16S rRNA gene abundance in sediments was distinctly higher in the low-salinity brackish sediments from HY (ranging from 2–8 × 10^7^ 16S rRNA gene copies cm^−3^) than the high-salinity marine sites at BR (2 × 10^4^–2 × 10^7^ 16S rRNA gene copies cm^−3^) and AR (4 × 10^6^–2 × 10^7^ 16S rRNA gene copies cm^−3^; Fig. 2). However, the proportions of *Archaea* increased with sediment depth from ∼1% at the sediment surface of all sites to 14.1, 7.5 and 2.3% of total prokaryotes at BR, AR and HY, respectively, suggesting that *Archaea* at the marine site BR, although having a lower abundance, were a larger fraction of the prokaryotic community (Fig. 2). This is consistent with findings reported for other estuarine and tidal flat sediments (Wilms *et al.*, 2007; Jiang *et al.*, 2011; Kubo *et al.*, 2012; Xie *et al.*, 2014).

### Archaea diversity in Colne Estuary sediments

#### Archaeal 16S rRNA gene diversity assessed by PCR cloning

Surface sediment samples at 2 cm depth (BR2, AR2 and HY2) were chosen for analysis of archaeal 16S rRNA gene diversity because they reflect most closely the salinity changes along the Colne Estuary (Figs 1 and 2). An additional sample at 30 cm was also analyzed from HY30, since this site had clear depth changes in chemical gradients (sulphate and methane) with a distinct methanogenic zone (Fig. 2c). Screening of *Archaea* 16S rRNA gene libraries by DNA hybridization with probe P958 (DeLong 1992) revealed that the majority (98–99%) of 1536 clones (384 clones per library) contained 16S rRNA gene inserts (Table 2). It should be noted that screening with probe P915 alone could have been misleading in that many clones (16–53%) containing *Archaea* 16S rRNA genes would not have been detected (Table 2). This highlights potential problems caused by primer/probe bias when targeting uncultivated lineages of *Archaea* in sediment samples (Teske and Sørensen 2008).

**Table 2.** DNA hybridization of Colne Estuary sediment archaeal 16S rRNA gene libraries (n = 384) with *Archaea*- and methanogen-specific oligonucleotide probes.

	% Clones hybridizing to oligonucleotide probe		
16S rRNA gene library^a^	P915-DIG *Archaea*	P958-DIG *Archaea*	P355-DIG *Methanosarcinales/Methanomicrobiales*
BR2	47	98	3
AR2	48	98	10
HY2	52	98	20
HY30	84	99	33

^a^BR2, BR 0–2 cm depth; AR2, AR 0–2 cm depth; HY2, HY 0–2 cm depth; HY30, HY 28–30 cm depth.

**Figure 3.**
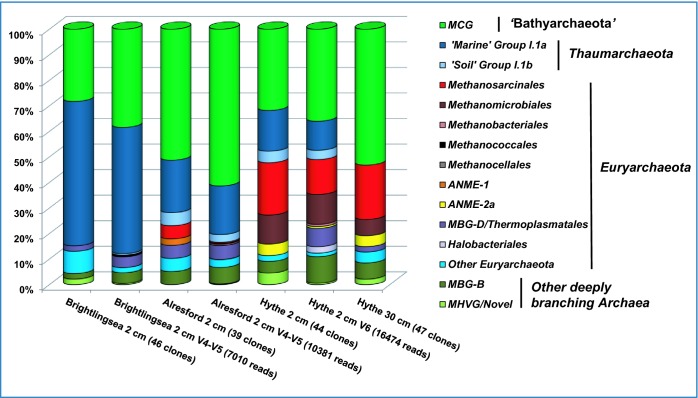
Diversity of archaeal 16S rRNA gene sequences from Colne Estuary sediments derived by PCR cloning (BR2, AR2, HY2 and HY30), V4–V5-tag sequencing (BR2 and AR2) and V6-tag sequencing (HY2). Numbers of clones or reads in each gene library are shown in parentheses.

Using P958-DNA hybridization as a guide, 50 clones were chosen at random from each library, and after exclusion of poor quality sequences, 39–47 clones from each sediment sample (total = 176 sequences) were used for estimating archaeal diversity (Figs 3 and 4; Table S1, Supporting Information). The archaeal sediment community at the high-salinity/high-sulphate estuary mouth (BR2) was dominated by the ‘marine’ group I.1a *Thaumarchaeota* and the candidate phylum ‘Bathyarchaeota’ (MCG), and at the low-salinity/low-sulphate estuary head (HY2) by methanogenic *Euryarchaeota* and MCG with fewer *Thaumarchaeota*. The archaeal community at AR2 seemed to reflect its location along the River Colne Estuary; having a high frequency of MCG, slightly lower numbers of *Thaumarchaeota* and fewer methanogens. Interestingly, in deeper sediments at HY30, no *Thaumarchaeota*-like sequences were found and the archaeal community was dominated by MCG and methanogenic *Euryarchaeota*.

**Figure 4.**
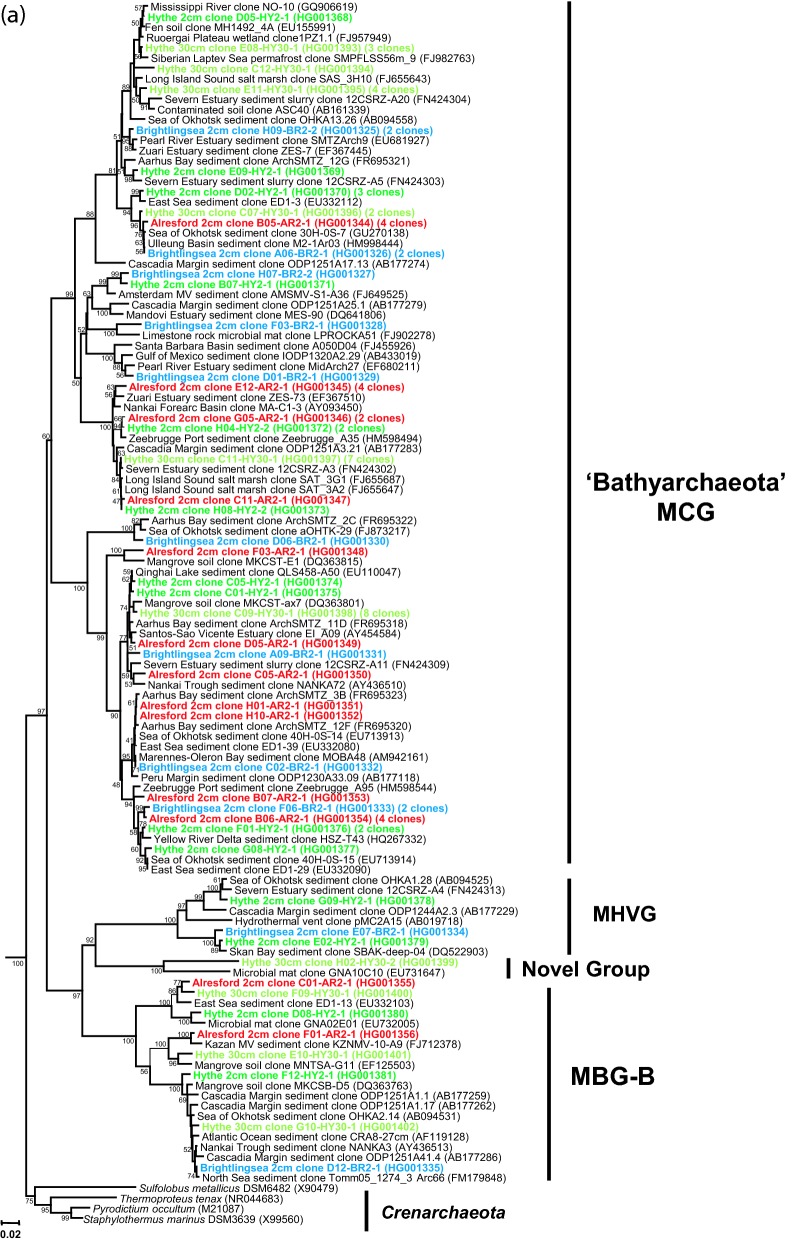
Phylogenetic trees showing the relationship of archaeal 16S rRNA gene sequences derived from Colne Estuary sediments to their nearest environmental and pure culture sequences. (a) *Crenarchaeota*, ‘Bathyarchaeota’ and other deeply branching *Archaea* (b) *Thaumarchaeota* (c) *Euryarchaeota*; trees were constructed with 600, 855 and 475 bases, respectively, of aligned 16S rRNA gene sequences. All trees were obtained using Minimum Evolution and LogDet distance and representative sequences of the *Korarchaeota* were used as out groups; clone SRI-306 (AF255604) and clone pJP27 (L25852). Bootstrap support values over 50% (1000 replicates) are shown. Sequences retrieved in this study are shown in bold and colour coded according to 16S rRNA gene library: blue, BR2; red, AR2; green, HY2; light green, HY30.

**Figure 4.**
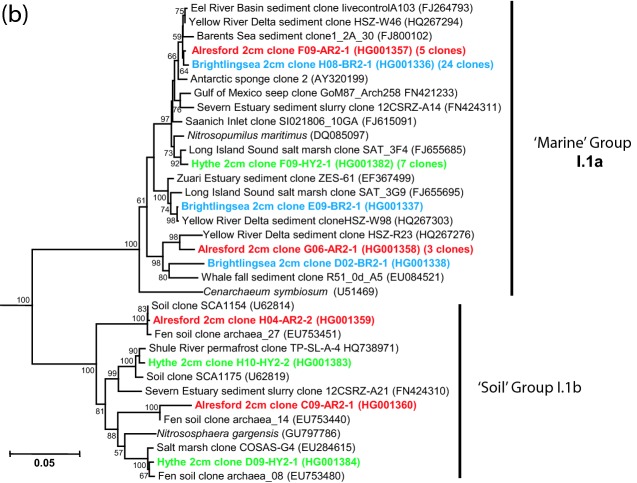
*Continued*.

**Figure 4.**
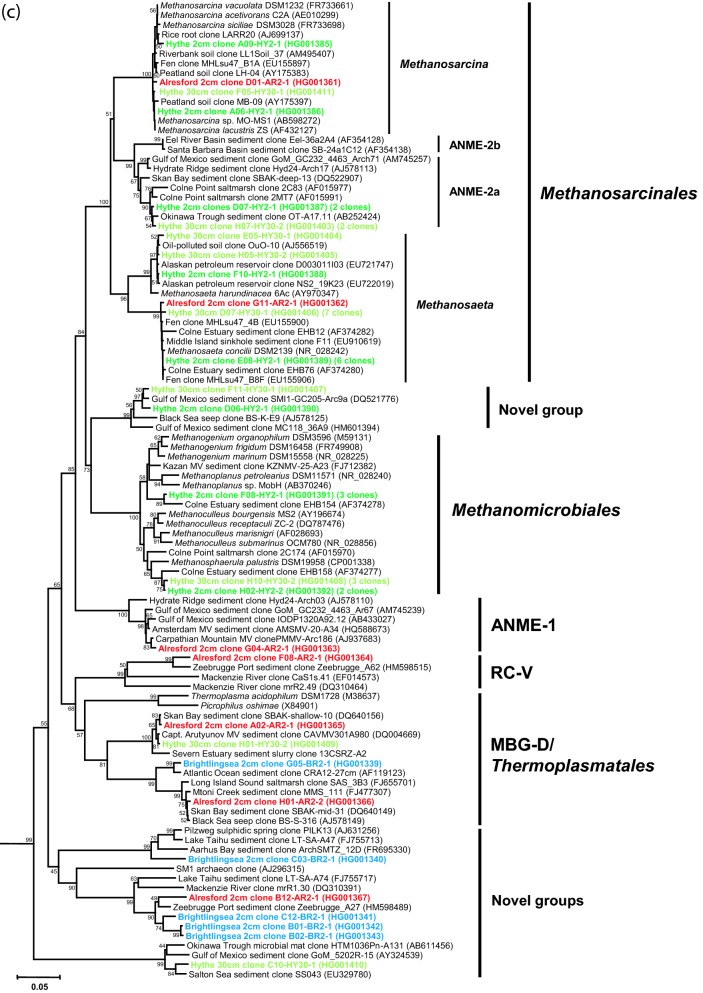
*Continued*.

Rarefaction curves, coverage estimates and estimators of species richness (S_Chao1_ and S_ACE_) indicated that the archaeal 16S rRNA gene libraries for each site were not sampled completely to capture the total estimated species richness (Table 3; Fig. S2, Supporting Information). However, all parameters suggest that the estuary mouth surface sediment site, BR2, has fewer archaeal OTUs compared with AR2 and HY2. The deeper sediment site HY30 also showed a reduced species richness, when compared with HY2. This difference in species richness was further supported by Shannon's and Simpson's indices of diversity (Table 3), which also suggested high archaeal diversity at AR2. High archaeal 16S rRNA gene diversity at this mid-estuary site could be due to the location and dynamic conditions influenced by both marine and freshwater inputs. Similar observations of high archaeal diversity were found in sediments from the Mandovi Estuary and a tidal marsh in south-eastern Connecticut; both influenced by strong tides and elevated land drainage (Nelson, Moin and Bernhard 2009; Singh *et al.*, 2010), as well as sediments from mid-locations in the Pearl River Estuary (Xie *et al.*, 2014). In addition, high diversity at AR could be associated with the high numbers of diverse MCG sequences at this site (Fig. 4a), possibly indicating a high degree of metabolic diversity (Kubo *et al.*, 2012; Lloyd *et al.*, 2013b) necessary for such dynamic conditions.

**Table 3.** Diversity indices for Colne Estuary sediment *Archaea* 16S rRNA and *mcr*A gene libraries using genus and species-level groupings (% similarity).

Gene library	Number of	Unique OTUs	Good's	Simpson's	Shannon's	*S*_Chao1_	*S*_ACE_
(% similarity)	clones		coverage (%)	diversity index (1-*D*)	diversity index (*H’*)		
**16S rRNA**							
BR2 (97)	46	20	65	0.71	2.08	50.79	72.57
BR2 (95)		17	72	0.69	1.90	42.23	60.60
AR2 (97)	39	27	44	0.94	3.10	103.20	109.76
AR2 (95)		22	57	0.92	2.80	91.18	72.50
HY2 (97)	44	27	55	0.94	3.05	74.98	85.90
HY2 (95)		24	66	0.93	2.95	45.74	51.18
HY30 (97)	47	19	77	0.90	2.60	36.61	37.20
HY30 (95)		17	79	0.89	2.43	32.19	34.21
*mcr*A							
BR2 (89)	37	8	95	0.74	1.63	8.25	10.36
AR2 (89)	30	12	77	0.85	2.16	33.65	23.18
HY2 (89)	33	10	82	0.69	1.65	17.95	21.44
HY30 (89)	28	9	78	0.71	1.61	17.71	32.40
**16S rRNA V6-tag**							
HY2 (97)	16474	259	98	0.96	5.39	328.04	ND
HY2 (94)		217	99	0.95	5.20	259.30	ND
**16S rRNA V4–V5-tag**							
BR2 (97)	7010	216	99	0.76	3.61	241.00	255.66
BR2 (94)		133	99	0.73	3.05	151.86	157.73
AR2 (97)	10381	327	99	0.89	4.56	334.25	346.14
AR2 (94)		200	99	0.86	3.74	205.45	214.30

BR2, BR 0–2 cm depth; AR2, AR 0–2 cm depth; HY2, HY 0- 2 cm depth; HY30, HY 28–30 cm depth.

OTU, operational taxonomic unit; ND, not determined.

*S*_Chao1_ and *S*_ACE_ represent the expected number of OTUs present in an environment if sampling were complete.

Shannon's and Simpson's indices are measures of species diversity and both increase with increasing genetic diversity.

#### Archaeal 16S rRNA gene diversity by tag sequencing

To compare the large-fragment 16S rRNA gene library results with alternative sequencing approaches with higher sequence throughput and different variable regions of the 16S rRNA gene, 16474 archaeal V6 16S rRNA gene tags were analyzed from HY2, and 7010 and 10381 archaeal V4-V5 16S rRNA gene tags were analyzed from BR2 and AR2, respectively, (Fig. 3) with a sample coverage of 98–99% at the species level (Table 3). Taxonomic assignments suggested that the overall archaeal community structure in Colne Estuary sediments at the phylum/major group level was already well represented by sequencing of 39–44 random clones, since the taxonomic profile obtained by both methods of tag sequencing was similar to that by conventional PCR cloning of the V2–V5 region (Fig. 3). However, some additional *Euryarchaeota* groups were identified by tag sequencing belonging to the *Halobacteriales* (0.03–2.5%) and the methanogen orders *Methanobacteriales* (0.2–0.9%), *Methanococcales* (0.01–0.02%) and *Methanocellales* (0.01%). Similar good agreement between *Archaea* phylum/major group level profiles obtained by tag sequencing and PCR cloning was also reported for other sedimentary environments including Gulf of Mexico seeps (Lloyd *et al.*, 2010) and Guaymas Basin hydrothermal sediments (Biddle *et al.*, 2011), suggesting that within marine sediments at least, the full range of major *Archaea* phyla and groups are already well represented in molecular surveys. Further analysis of the 16S rRNA gene tags revealed that, although the overall diversity at the phylum level was similar, large differences in diversity at the species and genus level were apparent (Table 3). For example, the number of unique archaeal OTUs estimated by 16S rRNA gene tag sequences was ∼10-fold higher than by PCR cloning and this high species richness, detected by tag sequencing, was supported by all diversity estimates (Table 3) highlighting that River Colne estuarine sediments have a much greater archaeal species richness than previously reported (Munson, Nedwell and Embley 1997). It should be noted that such direct comparisons of *Archaea* species richness and diversity using datasets derived by different 16S rRNA gene PCR primers should be treated with caution, as they may have different amplification biases. However, it has been shown that apart from regions V1–V2, taxonomic comparisons of other 16S rRNA variable regions are comparable, and metagenomic analyses do not indicate significant discrepancies with PCR-derived databases (Yarza *et al.*, 2014).

### Major archaeal phyla of the Colne Estuary

#### ‘Bathyarchaeota’

Detailed phylogenetic analysis of the archaeal 16S rRNA gene (V2–V5) libraries (Fig. 4) revealed that the majority of the *Archaea* in the Colne Estuary belonged to clades with no cultured isolates, although representatives of these groups are common in molecular surveys of marine sediments (Fry *et al.*, 2008; Teske and Sørensen 2008). Members of the newly proposed deeply branching phylum ‘Bathyarchaeota’ or MCG (Meng *et al.*, 2014), formerly of the *Crenarchaeota*, were the most abundant of all archaeal phyla in the Colne sediment 16S rRNA gene libraries (41% by PCR cloning, 49% by V4–V5-tag sequencing and 36% by V6-tag sequencing; Figs 3 and 4a). All MCG were widespread throughout the sediment sites and their presence did not relate to any identifiable geographical or environmental condition measured within this study.

The ‘Bathyarchaeota’ or MCG comprises a large number of phylogenetically diverse phylotypes from anoxic environments that can be split into 17 subgroups (Kubo *et al.*, 2012), and recently phylogenomic evidence has shown MCG to branch separately from the *Crenarchaeota* (Fig. 4a) and locate at a deep branching position with the *Thaumarchaeota* and ‘Aigarchaeota’ (Guy and Ettema 2011; Lloyd *et al.*, 2013b; Meng *et al.*, 2014). The broad range of habitats in which MCG phylotypes have been reported, including terrestrial palaeosol, freshwater lakes, marine sediments, hot springs and hydrothermal vents (Teske and Sørensen 2008), indicates the versatility of this group, and is consistent with them dominating the overall Colne Estuary sediment archaeal community. The characteristics that result in such dominance by MCG species are unknown, although recent evidence obtained by single cell genomics has shown that some members of the MCG degrade detrital proteins in subsurface sediments (Lloyd *et al.*, 2013b), compounds that are abundant in River Colne sediments (Agedah *et al.*, 2009). Some MCG have also been shown to incorporate ^13^C-acetate by DNA-SIP in sediments from the Severn Estuary (Webster *et al.*, 2010), supporting other reports indicating that they are heterotrophic and utilize buried organic carbon (Biddle *et al.*, 2006). Such findings are consistent with them being detected as a major component in other organic-rich estuarine sediments (Roussel *et al.*, 2009; Jiang *et al.*, 2011). In addition, Meng *et al.* (2014) reported that genes involved in protocatechuate degradation were present in a MCG fosmid, and subsequent expression of a putative 4-carboxymuconolactone decarboxylase in sediment microcosms supplemented with protocatechuate suggested that some MCG degrade aromatic compounds.

#### Thaumarchaeota

Overall, the *Thaumarchaeota* represented 25% of archaeal 16S rRNA gene sequences from Colne Estuary sediments and 29% of tags (Fig. 3), with the majority of sequences clustering within the ‘marine’ group I.1a (alternatively called MG-I; Teske and Sørensen 2008). However, in contrast to the ‘Bathyarchaeota*’*, phylogenetic analysis of *Thaumarchaeota* sequences (Fig. 4b) suggest that this phylum's distribution may be linked to changes in sediment depth, location and/or salinity gradient along the estuary. For example, *Thaumarchaeota* sequences were only in surface (2 cm) sediments (i.e. absent in HY30), all *Thaumarchaeota* sequences from BR2 belonged to the ‘marine’ group I.1a, and no ‘soil’ group I.1b were found in this high-salinity/sulphate (marine) environment by PCR cloning (only 0.5% of V4–V5 tags). Sequences belonging to ‘soil’ group I.1b were primarily in sediments with reduced salinity/sulphate (AR and HY; Figs 3 and 4b), whereas ‘marine’ group I.1a were present at all sites. In addition, sequences of ‘marine’ group I.1a reduce in frequency away from the estuary mouth as salinity decreases, representing 49–57% of 16S rRNA gene sequences and tags in BR2, 11–19% in AR2 and 11–20% in HY2 samples.

*Thaumarchaeota*, ubiquitous in marine and freshwater, soils and sediments, represent a large prokaryotic biomass involved in nitrification (Wuchter *et al.*, 2006; Prosser and Nicol 2008). To date, all cultured representatives of *Thaumarchaeaota* are aerobic autotrophic ammonia oxidizers (Könneke *et al.*, 2005; Tourna *et al.*, 2011), accounting for their unique distribution within the surface sediments of the Colne Estuary. In addition, the dominance of ‘marine’ group I.1a at BR may also be explained by cultured representatives of this group having an high affinity for ammonia (Könneke *et al.*, 2005; Tourna *et al.*, 2011), an important factor in Colne Estuary marine sediments that have low concentrations of ammonia (Dong *et al.*, 2000; Thornton *et al.*, 2002). Salinity has also been emphasized as being an important factor governing the spatial distribution of ammonia oxidizers in other estuarine environments (Sahan and Muyzer 2008), and often the water column/sediment *amoA* group (equivalent to ‘marine’ group I.1a) are the most abundant archaeal *amoA* genes in estuarine sediments (Bernhard and Bollmann 2010). Similarly, the present study provides strong evidence that ‘marine’ group I.1a are dominant in high-salinity marine sediments, whereas, the ‘soil’ group I.1b are found less frequently and only detected in areas of the estuary which have a strong influence of freshwater and soil run-off, similar to that observed by Dang *et al.* (2008) in the Changjiang Estuary. Although salinity is often identified as a key factor in regulating ammonia oxidizer community composition and abundance (Sahan and Muyzer 2008), it is probable that it is not the only factor. For example, *Archaea* ammonia oxidizer abundance has also been related to pH, clay content, heavy metals and sulphide concentrations, factors which often co-vary with salinity (Bernhard and Bollmann 2010). Alternatively, the reduction in *Thaumarchaeota* 16S rRNA genes at AR2 and HY2 (Fig. 3) may be linked with increased ammonia in surface sediments at the estuary head (Thornton *et al.*, 2002), as it is known that *Betaproteobacteria* ammonia oxidizers out-compete *Archaea* ammonia oxidizers under high ammonia conditions (Bouskill *et al.*, 2011) and that *amoA* genes in the Colne Estuary are dominated by *Betaproteobacteria* ammonia oxidizers (Li *et al.*, 2014).

#### Euryarchaeota

Sequences belonging to the *Euryarchaeota* comprised 27% of all archaeal 16S rRNA genes by PCR cloning and 18% by tag sequencing, with at least 10 distinct major taxa (Figs 3 and 4c) and four potentially new clades (Fig. 4c).

Apart from methanogens (see below), *Euryarchaeota* sequences in the marine sediments of BR2 belonged to uncultivated groups and were either MBG-D/*Themoplasmatales* or novel groups loosely associated (<80% sequence similarity) with SM1 *Archaea* found in cold sulphidic springs (Rudolph *et al.*, 2004). Recently, some single cell genomes of MBG-D have shown them to contain genes that encode extracellular protein-degrading enzymes that could enable them to survive on sedimentary detrital proteins (Lloyd *et al.*, 2013b). Similarly, MBG-D have been maintained in heterotrophic enrichment cultures from sediments of Aarhus Bay (Webster *et al.*, 2011). Whereas, other reports suggest that some members of the *Thermoplasmatales* and related *Euryarchaeota* lineages may represent a novel order of methanogens (Paul *et al.*, 2012; Borrel *et al.*, 2013) that can utilize methylamine (Poulsen *et al.*, 2013). Novel *Euryarchaeota* sequences were also present at HY and AR, but these were often the minority, as were sequences belonging to Rice Cluster V (RC-V), MBG-D and the anaerobic methanotrophic *Archaea* (ANME) groups ANME-1 and 2a (Figs 3 and 4c). The ANME are a diverse group of *Euryarchaeota* related to the methanogen orders *Methanosarcinales* and *Methanomicrobiales* which gain energy exclusively from anaerobic oxidation of methane (AOM) coupled with bacterial sulphate reduction (Knittel and Boetius 2009).

*Methanosarcinales* and *Methanomicrobiales* were the most abundant methanogen groups (e.g. 16% of all clones, 25% of 16S rRNA gene tags at HY2) and representatives of these orders increased in frequency towards the estuary head (Fig. 3; Table S1, Supporting Information). For example, few methanogen 16S rRNA gene phylotypes were present at BR2 (only 0.3% and V4–45 tags belonged to *Methanosarcinales*), but sequences related to *Methanosarcina*, *Methanosaeta* (*Methanosarcinales*), *Methanogenium*, *Methanoculleus* (*Methanomicrobiales*) and a novel *Methanomicrobiales*-related group were numerous in libraries from brackish sediments at HY (HY2 and HY30). Relatively low numbers of *Methanosarcinales*/*Methanomicrobiales* sequences and tags were obtained from the mid-estuary site AR (Fig. 3). Hybridization of the *Archaea* 16S rRNA gene libraries with the specific *Methanosarcinales/Methanomicrobiales* probe P335 (Table 2) clearly confirmed the increased abundance of methanogens towards the estuary head, and with increasing depth at HY (Table 2). This correlates with the increasing methane concentrations (Fig. 2c) and rates of methanogenesis (O'Sullivan *et al.*, 2013). High numbers of methanogens at HY supports previous findings that anaerobic terminal organic carbon degradation in Colne Estuary sediments changes from being dominated by sulphate reduction at the marine end to being methanogenesis-driven at the freshwater head (Nedwell, Embley and Purdy 2004). This is presumably due to reduced competition for electron donors with sulphate limitation (Liu and Whitman 2008) and the reported increase in DOC (Thornton *et al.*, 2002), providing a range of substrates to support a metabolically diverse population of methanogens.

**Figure 5.**
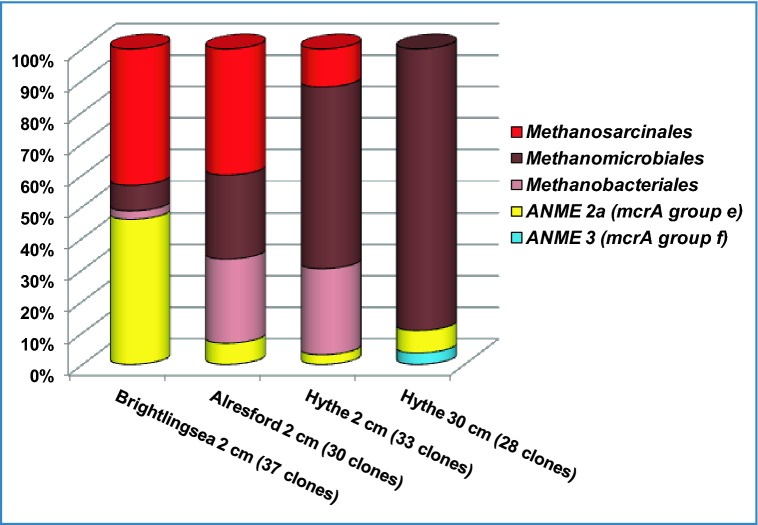
Diversity of *mcr*A gene sequences from Colne Estuary sediments derived by PCR cloning (BR2, AR2, HY2 and HY30). Numbers of clones in each gene library are shown in parentheses.

The presence of a diverse population of methanogens within the Colne Estuary was confirmed by analysis of *mcr*A genes. All diversity parameters for *mcr*A gene libraries suggested a higher level of coverage (77–95%) and *mcr*A gene diversity was low (Table 3), although at AR, *mcr*A gene diversity was higher than at the other two sites. The majority of *mcr*A sequences (Fig. 5; Table S2, Supporting Information) in Colne Estuary sediments were assigned to *Methanosarcinales*, *Methanomicrobiale*s, *Methanobacteriales* and the closely related methanotrophic ANME *mcr*A group e (thought to be ANME-2a; Knittel and Boetius 2009). Methanogen *mcr*A gene phylotypes increased in frequency with respect to a decrease in ANME *mcr*A gene phylotypes (Fig. 5) as salinity and sulphate concentrations decreased away from the estuary mouth (Fig. 2; 54% at BR, 89–97% at HY). This increase in methanogen *mcr*A gene phylotypes coincided with the observed increase in methanogen 16S rRNA genes towards the estuary head (Fig. 3; Table 2). Whereas, the decrease in the number of ANME *mcr*A sequences (Fig. 5) was probably linked to the methanotrophic *Archaea* being associated with marine sediments and sulphate-dependent AOM (Knittel and Boetius 2009).

#### Other deeply branching Archaea

Small numbers of Marine Benthic Group-B (MBG-B) and the Marine Hydrothermal Vent Group (MHVG) were also found in Colne Estuary sediments (Fig. 4a). Members of these two deeply branching groups of *Archaea* have previously been identified in estuarine sediments (Webster *et al.*, 2010; Jiang *et al.*, 2011). Isotopic data from archaeal cell membranes suggests that MBG-B can assimilate recalcitrant carbon (Biddle *et al.*, 2006), while other studies propose that MBG-B benefit directly or indirectly from methane cycling (Inagaki *et al.*, 2006; Teske and Sørensen 2008). This association with methane cycling could account for the slight increase in their frequency within sediments at HY, which have increased methane, high methanogenesis and evidence of AOM (Fig. 2c; O'Sullivan *et al.*, 2013) and high organic carbon (Thornton *et al.*, 2002).

### *Methanosarcinales* and *Methanomicrobiales* are important members of the Colne Estuary

Several reports have shown that members of the *Methanosarcinales* and *Methanomicrobiales* are the most commonly found methanogens in estuarine sediments (Purdy *et al.*, 2002; Banning *et al.*, 2005; Jiang *et al.*, 2011; Li *et al.*, 2012; O'Sullivan *et al.*, 2013; Chen *et al.*, 2014), and in this study sequences (16S rRNA and *mcr*A genes) belonging to these two orders were the predominant methanogen phylotypes throughout the Colne Estuary sediments. *Methanosarcinales* and *Methanomicrobiales* are often found together apparently because members of these two orders differ in their substrate utilization (Liu and Whitman 2008). Generally, *Methanomicrobiales* only use H_2_/CO_2_ as a substrate for methanogenesis, while members of the *Methanosarcinales* can utilize a number of different substrates (e.g. *Methanosarcina* utilize H_2_/CO_2_, methyl compounds and/or acetate, *Methanosaeta* use acetate and *Methanococcoides* utilize methyl compounds (Ferry 2010).

In estuarine sediments along a salinity/sulphate gradient, the availability of specific methanogen substrates can vary due to competition from sulphate-reducing bacteria (SRB) resulting in methanogen populations being niche partitioned depending on their substrate usage (Purdy *et al.*, 2002). For example, all members of the *Methanosarcinales* identified at BR by *mcr*A gene sequencing (Fig. 5; Table S2, Supporting Information) belonged to *Methanococcoides*, *Methanolobus* and *Methanosarcina*, species that are able to utilize non-competitive substrates, such as methanol and methylated amines that most SRB cannot use (Oremland, Marsh and Polcin 1982). 16S rRNA gene qPCR of the methylotrophic *Methanococcoides* species also showed that these methanogens were much more abundant in the top 10 cm and constituted a larger fraction of the overall archaeal population at BR (Fig. 2a) than at HY (Fig. 2c), and this is supported by previous studies in which *Methanococcoides* were readily detectable at BR and nearby Colne Point (Purdy *et al.*, 2002; O'Sullivan *et al.*, 2013). Interestingly, at BR (and AR) *Methanococcoides* 16S rRNA genes (100% sequence similarity to *M. burtonii*; Table S3, Supporting Information) progressively increased as a proportion of the *Archaea* (2–20%) with depths down to 10 cm (Fig. 2), after which their abundance rapidly declined to < 1% of *Archaea*. This supports that their presence directly relates to higher availability of non-competitive methylated substrates near the sediment surface (King 1984). Furthermore, the *Methanococcoides* qPCR depth profiles in this study match closely the changes in methanogen DGGE patterns presented in O'Sullivan *et al.* (2013). These first estimates of the abundance of *Methanococcoides* species (0.02–1.5% of prokaryotes; Fig. 2) in estuarine sediments clearly demonstrate that they represent a significant population and suggest that methylotrophic methanogenesis may contribute more to methane and nitrogen cycling in marine sediments than previously thought (Ferry and Lessner 2008).

In the low-salinity/sulphate sediments at HY the majority of *Methanosarcinales* 16S rRNA genes were closely related to the acetotrophic methanogens, *Methanosaeta concilii* and *M. harundinacea*. However, no *Methanosaeta*-like *mcr*A genes were found at HY; instead *Methanosarcinales mcr*A sequences belonged to *Methanosarcina* (93% sequence similarity to *Methanosarcina mazei*). Such inconsistencies in the frequency of observed marker genes for the same archaeal group may reflect their low abundance within the archaeal community or biases imposed by different gene primers and/or from the use of nested PCR. Recently, specific *mcr*A and 16S rRNA gene primers and repeated PCR amplifications have been used to study the ecology of *Methanosaeta* in the Colne Estuary (Carbonero *et al.*, 2012; Oakley *et al.*, 2012). However, despite these discrepancies, the detection of methanogens that can utilize acetate (*Methanosaeta* and *Methanosarcina*) and the detection of low acetate concentrations (Fig. S1, Supporting Information), and high rates of acetotrophic methanogenesis (O'Sullivan *et al.*, 2013) supports findings that acetate could be an important substrate for methanogenesis in low-salinity estuarine sediments (Purdy *et al.*, 2002, 2003; O'Sullivan *et al.*, 2013). HY sediments also contained large numbers of novel *Methanomicrobiales mcr*A gene sequences (Fig. 5; Table S2, Supporting Information) assigned to the so-called ‘Fen Cluster’ (Galand *et al.*, 2002), which increased with depth (58% of HY2 and 89% of HY30). These *mcr*A gene sequences are often associated with freshwater environments, such as river bank soils, peats and oligotrophic fens (Galand *et al.*, 2002; Conrad *et al.*, 2008; Steinberg and Regan 2008) and are related to the hydrogenotrophic methanogen *Methanoregula boonei* isolated from an acidic peat bog (Bräuer *et al.*, 2006). *Methanomicrobiales* 16S rRNA genes closely related to other hydrogenotrophic *Methanoplanus* and *Methanosphaerula* were also identified (Fig. 4c) and coupled with the *Methanomicrobiales mcr*A genes supports the relatively high rates of hydrogenotrophic methanogenesis previously reported at this site (O'Sullivan *et al.*, 2013). Curiously, high sulphate reduction rates were also present in HY sediments (O'Sullivan *et al.*, 2013), despite low concentrations of sulphate (Fig. 2c), and this is thought to be due to SRB populations that are able to respond rapidly to the occasional tidal incursion (Purdy *et al.*, 2003; O'Sullivan *et al.*, 2013). However, since this site has generally low concentrations of sulphate and high concentrations of organic matter (Thornton *et al.*, 2002), it provides conditions that are suitable for the co-existence of competitive methanogenesis and sulphate reduction (Oremland and Polcin 1982).

Interestingly, surface sediments at AR, which had lower rates of methanogenesis than HY, but higher rates than surface sediments at BR (O'Sullivan *et al.*, 2013), contained a mixture of methylotrophic (*Methanosarcina*, *Methanococcoides*), acetotrophic (*Methanosarcina*, *Methanosaeta*) and hydrogenotrophic (novel *Methanomicrobiales* ‘Fen cluster’) methanogens, as well as some *Methanobacteriales* sequences related to the hydrogenotrophic *Methanobrevibacter* (Fig. 5; Table S2, Supporting Information). This may provide further indication that archaeal populations at AR are a reflection of their mid-estuarine position.

## SUMMARY

River Colne estuarine sediments are hypernutrified and contain a diverse population of *Archaea*, represented throughout by phylotypes from all of the main phyla, with many sequences from novel and uncultivated lineages, and some assigned groups with known or putative physiologies; e.g. methanogens, methanotrophs (*Euryarchaeota*), ammonia oxidizers (*Thaumarchaeota*) and heterotrophic protein degraders (‘Bathyarchaeota*’* MCG). Some archaeal lineages, notably the MCG, are widespread throughout the estuary, whereas others (e.g. methanogens and ammonia oxidizers) are more localized, and may have been selected for by specific conditions along the estuarine gradient. For example, clear differences between the marine and brackish archaeal communities are evident, comparing estuary mouth (BR) and estuary head (HY) sediments. This difference in *Archaea* composition suggests niche separation linked to differences in salinity, sulphate, organic carbon and ammonia gradients (Thornton *et al.*, 2002; Xie *et al.*, 2014). More specifically, results presented here show that the composition of *Thaumarchaeota* varied with salinity, as only ‘marine’ group I.1a was found in marine sediments (BR) and that methanogenic *Euryarchaeota* (16S rRNA and *mcr*A phylotypes) increased proportionally with decreasing salinity and sulphate gradients. Methanogen populations in brackish sediments (HY) are dominated by obligately hydrogenotrophic and acetoclastic (*Methanosaeta*) methanogen types, with a few potentially versatile *Methanosarcina* species. Conversely, marine surface sediments (BR) had a high proportion of *Methanococcoides*, *Methanolobus* and *Methanosarcina* species, which are all able to utilize non-competitive methyl substrates. This study extends our understanding of some of the important environmental factors that structure archaeal assemblages under natural conditions and suggests that salinity and other associated factors may be a significant feature controlling the distribution and abundance of estuarine sediment *Archaea*.

## SUPPLEMENTARY DATA

Supplementary data is available at FEMSEC online.

Supplementary data is available at FEMSEC online
