# Supplementary material for: Archaeal community diversity and abundance changes along a natural salinity gradient in estuarine sediments
Source: FEMS Microbiol Ecol. 2014 Dec 15;91(2):1–18. doi: 10.1093/femsec/fiu025 (PMC4399439; doi:10.1093/femsec/fiu025)
Supplement: Supplementary data is available at FEMSEC online [file femsec_fiu025_index.html]

SUPPLEMENTARY DATA | FEMS Microbiology Ecology

## SUPPLEMENTARY DATA

**Files in this Data Supplement:**

- Figure S1.
