# Supplementary material for: Archaeal community diversity and abundance changes along a natural salinity gradient in estuarine sediments
Source: FEMS Microbiol Ecol. 2014 Dec 15;91(2):1–18. doi: 10.1093/femsec/fiu025 (PMC4399439; doi:10.1093/femsec/fiu025)
Supplement: Supplementary data is available at FEMSEC online [file supplementary_tables_figs.doc]

**Figure S1** Depth profiles of pore water volatile fatty acid (acetate, lactate and formate) concentrations for Colne Estuary sediment cores.

**Brightlingsea (BR)**

**Alresford (AR)**

**Hythe (HY)**

**Figure S2** Rarefaction curves for the 16S rRNA and *mcrA* gene libraries from the Colne Estuary sediment sites. Curves were plotted for (a) 97% and (b) 95% similarity for 16S rRNA gene libraries and (c) 89% similarity for *mcrA* gene libraries, representing approximately the species and genus level.

**(a)**

**(b)**

**Figure S2 continued**

**(c)**

**Table S1** Relative abundance (% library) of *Archaea* groups in 16S rRNA gene libraries from Colne Estuary sediments derived by PCR-cloning (BR2, AR2, HY2 and HY30), V4-V5-tag sequencing (BR2 and AR2) and V6-tag sequencing (HY2).

| Phylum | Group | Relative abundance of *Archae*a Groups (% library) | | | | | | |
| --- | --- | --- | --- | --- | --- | --- | --- | --- |
|  |  | 16S rRNA gene clone library V2-V5 | | | | 16S rRNA gene V4-V5 | | 16S rRNA gene V6 |
|  |  | BR2 | AR2 | HY2 | HY30 | BR2 | AR2 | HY2 |
| ***Thaumarchaeota*** | **'Marine' Group I.1a** | 56.5 | 20.5 | 16 | - | 49.2 | 19 | 11.4 |
|  | **'Soil' Group I.1b** | - | 5.1 | 4.5 | - | 0.6 | 3 | 3.6 |
| *‘*Bathyarchaeota*’* | MCG | 28.3 | 51.4 | 31.8 | 53.2 | 38.3 | 61 | 36 |
| Other *Archaea* | MBG-B | 2.2 | 5.1 | 4.5 | 6.4 | 4.2 | 6.4 | 10.4 |
|  | MHVG/Novel | 2.2 | 0 | 4.5 | 2.1 | 0.5 | 0.3 | 0.6 |
| *Euryarchaeota* | *Methanosarcinales* | - | 5.1 | 20.5 | 21.2 | 0.3 | 0.4 | 13.7 |
|  | *Methanomicrobiales* | - | - | 11.5 | 6.4 | >0.1 | 0.3 | 11.7 |
|  | *Methanobacteriales* | - | - | - | - | 0.2 | 0.5 | 0.8 |
|  | *Methanococcales* | - | - | - | - | - | >0.1 | >0.1 |
|  | *Methanocellales* | - | - | - | - | - | - | >0.1 |
|  | ANME-1 | - | 2.6 | - | - | 0.2 | >0.1 | - |
|  | ANME-2 | - | - | 4.5 | 4.3 | - | - | 0.7 |
|  | *Halobacteriales* | - | - | - | - | >0.1 | 0.1 | 2.5 |
|  | MBG-D/*Thermoplasmatales* | 2.2 | 5.1 | - | 2.1 | 4.2 | 5.5 | 7.2 |
|  | Other *Euryarchaeota* | 8.6 | 5.1 | 2.2 | 4.3 | 2 | 3 | 1.4 |
|  |  |  |  |  |  |  |  |  |
| Number of clones/tags | | 46 | 39 | 44 | 47 | 7010 | 10381 | 16474 |

**Table S2** Methanogen *mcrA* gene sequence matches to clone sequences from Colne Estuary sediment.

| Clone library | Representative *mcrA* clone  (accession number) | Phylogenetic Group | | Number of clones | Nearest sequence match  (accession number) | % Sequence similarity |
| --- | --- | --- | --- | --- | --- | --- |
| BR2 |  | *Euryarchaeota* |  | 37 |  |  |
|  | A09-BR2-M (HG001415) | *Methanosarcinales* | *mcrA* group e  (ANME-2a) | 15 | Marennes-Oléron Bay sediment clone MOBOcr43649 (AM942092) | 97 |
|  | A01-BR2-M (HG001414) | *Methanosarcinales* | *Methanosarcina* | 10 | Pearl River Estuary sediment cloneTopMcrA32 (EU681941) | 93 |
|  | B09-BR2-M (HG001416) | *Methanosarcinales* | *Methanococcoides* | 4 | *Methanococcoides methylutens* (U22235) | 98 |
|  | E02-BR2-M (HG001417) | *Methanosarcinales* | *Methanolobus* | 2 | Eel River Basin sediment clone so4M70 (FJ264879) | 96 |
|  | D07-BR2-M (HG001418) | *Methanosarcinales* | *mcrA* group e  (ANME-2a) | 2 | Marennes-Oléron Bay sediment clone MOBOcr43972 (AM942094) | 89 |
|  | D12-BR2-M (HG001419) | *Methanomicrobiales* | *Methanogenium* | 2 | Pearl River Estuary sediment clone McrA2 (EU681934) | 90 |
|  | G01-BR2-M (HG001420) | *Methanomicrobiales* | - | 1 | Brackish lake sediment clone Beu4ME-51 (AY625601) | 91 |
|  | GO4-BR2-M (HG001421) | *Methanobacteriales* | *Methanobrevibacter* | 1 | *Methanobrevibacter smithii* (CP000678) | 98 |
| AR2 |  | *Euryarchaeota* |  | 30 |  |  |
|  | B01-AR2-M (HG001422) | *Methanosarcinales* | *Methanosarcina* | 8 | Marennes-Oléron Bay sediment clone MOBOcr43032 (AM942088) | 97 |
|  | A09-AR2-M (HG001423) | *Methanosarcinales* | - | 3 | Mangrove sediment clone M0A02_02 (GU395726) | 91 |
|  | A05-AR2-M (HG001424) | *Methanosarcinales* | *Methanococcoides* | 1 | *Methanococcoides methylutens* (U22235) | 98 |
|  | D07-AR2-M (HG001425) | *Methanosarcinales* | *mcrA* group e  (ANME-2a) | 1 | Marennes-Oléron Bay sediment clone MOBOcr43972 (AM942094) | 92 |
|  | A07-AR2-M (HG001426) | *Methanosarcinales* | *mcrA* group e  (ANME-2a) | 1 | Marennes-Oléron Bay sediment clone MOBOcr43649 (AM942092) | 99 |
|  | A08-AR2-M (HG001427) | *Methanomicrobiales* | Fen cluster | 6 | Peatland bog clone mcrA_bog_20 (EU980425) | 95 |
|  | B09-AR2-M (HG001428) | *Methanomicrobiales* | - | 1 | Brackish lake sediment clone Beu4ME-51 (AY625601) | 90 |
|  | D01-AR2-M (HG001429) | *Methanomicrobiales* | *Methanogenium* | 1 | Pearl River Estuary sediment clone McrA2 (EU681934) | 90 |
|  | A03-AR2-M (HG001430) | *Methanobacteriales* | *Methanobrevibacter* | 3 | *Methanobrevibacter smithii* (CP000678) | 98 |
|  | B05-AR2-M (HG001431) | *Methanobacteriales* | - | 3 | Solid waste bioreactor clone JME35 (FJ435848) | 97 |
|  | A06-AR2-M (HG001432) | *Methanobacteriales* | *Methanobrevibacter* | 1 | Sewer clone JPL-17 (EF628161) | 98 |
|  | F03-AR2-M (HG001433) | *Methanobacteriales* | *Methanobrevibacter* | 1 | *Methanobrevibacter smithii* (CP000678) | 99 |
| HY2 |  | *Euryarchaeota* |  | 33 |  |  |
|  | H01-HY2-M (HG001434) | *Methanosarcinales* | *Methanosarcina* | 2 | Florida everglades soil clone MCR-F1SU-25 (AY459316) | 95 |
|  | A05-HY2-M (HG001435) | *Methanosarcinales* | *Methanomethylovorans* | 1 | *Methanomethylovorans hollandica* (AY260442) | 99 |
|  | C06-HY2-M (HG001436) | *Methanosarcinales* | - | 1 | Mangrove sediment clone M0B11_03 (GU395798) | 91 |
|  | B03-HY2-M (HG001437) | *Methanosarcinales* | *mcrA* group e  (ANME-2a) | 1 | Pearl River Estuary sediment cloneTopMcrA5 (EU681937) | 94 |
|  | A02-HY2-M (HG001438) | *Methanomicrobiales* | Fen cluster | 17 | Rice root (river bank soil) clone LLm_ADT43_30 (AM746900) | 98 |
|  | F05-HY2-M (HG001439) | *Methanomicrobiales* | *Methanogenium* | 1 | Pearl River Estuary sediment clone McrA2 (EU681934) | 90 |
|  | C02-HY2-M (HG001440) | *Methanomicrobiales* | *-* | 1 | Brackish lake sediment clone Beu4L-3 (AY625578) | 96 |
|  | A10-HY2-M (HG001441) | *Methanobacteriales* | - | 5 | Solid waste bioreactor clone JME35 (FJ435848) | 98 |
|  | G05-HY2-M (HG001442) | *Methanobacteriales* | *-* | 3 | Rice root (river bank soil) clone LLm_ADT43_47 (AM746916) | 97 |
|  | B08-HY2-M (HG001443) | *Methanobacteriales* | *Methanobrevibacter* | 1 | *Methanobrevibacter smithii* (CP000678) | 99 |
| HY30 |  | *Euryarchaeota* |  | **28** |  |  |
|  | E01-HY30-M (HG001444) | *Methanosarcinales* | *mcrA* group e  (ANME-2a) | 1 | Marennes-Oléron Bay sediment clone MOBOcr43649 (AM942092) | 98 |
|  | A05-HY30-M (HG001445) | *Methanosarcinales* | *mcrA* group e  (ANME-2a) | 1 | Pearl River Estuary sediment cloneTopMcrA5 (EU681937) | 95 |
|  | B01-HY30-M (HG001446) | *Methanosarcinales* | *mcrA* group f  (ANME-3) | 1 | Marennes-Oléron Bay sediment clone MOBPc431232 (AM942106) | 86 |
|  | A10-HY30-M (HG001447) | *Methanomicrobiales* | Fen cluster | 13 | Peatland bog clone mcrA_bog_20 (EU980425) | 94 |
|  | C09-HY30-M (HG001448) | *Methanomicrobiales* | - | 7 | Humic bog clone 116 (GU084944) | 96 |
|  | F01-HY30-M (HG001449) | *Methanomicrobiales* | - | 2 | Brackish lake sediment clone Beu4ME-51 (AY625601) | 91 |
|  | C11-HY30-M (HG001450) | *Methanomicrobiales* | Fen cluster | 1 | Marennes-Oléron Bay sediment clone MOBOcr43968 (AM942093) | 91 |
|  | G11-HY30-M (HG001451) | *Methanomicrobiales* | Fen cluster | 1 | Rice root (river bank soil) clone LLm_ADT43_38 (AM746908) | 90 |
|  | B07-HY30-M (HG001452) | *Methanomicrobiales* | - | 1 | Humic bog clone 116 (GU084944) | 93 |

All *mcrA* gene sequences were assigned to phylotypes by using BLASTClust (http://www.ncbi.nlm.nih.gov/) at 89% cut-off (Steinberg & Regan, 2008)

**Table S3** *Methanococcoides* 16S rRNA gene sequence matches to clone sequences from Colne Estuary sediment derived from specific qPCR primers.

| Clone library | Representative *mcrA* clone (accession number) | Phylogenetic Group | | Number of clones | Nearest sequence match  (accession number) | % Sequence similarity |
| --- | --- | --- | --- | --- | --- | --- |
| BR2 |  | *Euryarchaeota* |  | 27 |  |  |
|  | Mc1-BR2 (HG001412) | *Methanosarcinales* | *Methanococcoides* | 26 | *Methanococcoides burtonii* DSM 6242 (NR_074242) | 100 |
|  | Mc14-BR2 (HG001413) | *Methanosarcinales* | *Methanococcoides* | 1 | *Methanococcoides methylutens* DSM2657 (FR733669) | 98 |
